# Anatomical and Biomechanical Stability of Single/Double Screw‐Cancellous Bone Fixations of Regan–Morry Type III Ulnar Coronoid Fractures in Adults: CT Measurement and Finite Element Analysis

**DOI:** 10.1111/os.13664

**Published:** 2023-01-16

**Authors:** Hao Ye, Yongchao Yang, Tingyang Xing, Guirong Tan, Shuxun Jin, Zhichao Zhao, Weikang Zhang, Yanyan Li, Lei Zhang, Jianshun Wang, Rongmei Zheng, Yun Lu, Lijun Wu

**Affiliations:** ^1^ Institute of Digitized Medicine and Intelligent Technology Wenzhou Medical University Wenzhou China; ^2^ Department of Orthopedics Tianjin Teda Hospital Tianjin China; ^3^ Department of Orthopedics, The Third Affiliated Hospital Wenzhou Medical University Wenzhou China; ^4^ Department of Orthopedics, The Second Affiliated Hospital Wenzhou Medical University Wenzhou China

**Keywords:** Anatomical and Biomechanical Stability, Digital Orthopaedics Surgery, Finite Element Method, Smart Screw Internal Fixation, Ulnar Coronoid Fracture

## Abstract

**Objective:**

At present, it is still uncertain whether single screw has the same stability as double screws in the treatment of ulnar coronal process basal fracture (Regan–Morry type III). So, we aimed to compare the pull‐out force and anti‐rotation torque of anterior single/double screw‐cancellous bone fixation (aSSBF, aDSBF) in this fracture, and further study the influencing factors on anatomical and biomechanical stability of smart screw internal fixations.

**Methods:**

A total of 63 adult volunteers with no history of elbow injury underwent elbow CT scanning with associated three‐dimensional reconstruction that enabled the measurements of bone density and fixed length of the proximal ulna and coronoid. The models of coronal process basal fracture, aSSBF and aDSBF, were developed and validated. Using the finite element model test, the sensitivity analysis of pull‐out force and rotational torque was carried out.

**Results:**

The pull‐out force of aSSBF model was positively correlated with the density of the cancellous bone and linearly related to the fixed depth of the screw. The load pattern of pull‐out force of aDSBF model was similar to that of aSSBF model. The ultimate torque of aDSBF model was higher than that of aSSBF model, but the load pattern of ultimate torque of both models was similar to each other when the fracture reset was satisfactory, and the screw nut attaches closely to coronoid process. Moreover, with enhancement of initial pre‐tightening force, the increase of ultimate torque of both models was small.

**Conclusions:**

In addition to three pull‐out stability factors of smart screw fixations, fracture surface fitting degree and nut fitting degree are the other two important anatomical and biomechanical stability factors of smart screw fixations both for rotational stability. When all pull‐out stability and rotational stability factors meet reasonable conditions simultaneously, single or double screw fixation methods are stable for the treatments of ulnar coronoid basal fractures.

## Graphical abstract

Biomechanics of 12 finite element (FE) models of fractured and surgical elbows were simulated. The pull‐out force and rotation torque of screw fixations were ranked by finite element model (FEM). Two new stability factors of screw fixations were determined by FEM sensitivity test. Three‐dimensional anatomy and biomechanical FEM contribute to orthopaedic optimization.

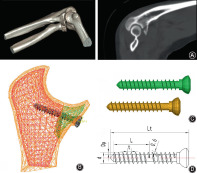

## Introduction

The ulnar coronoid is an important skeletal structure in front of the elbow joint.^1^, ^2^ Coronoid fractures result in loss of skeletal stability and are associated soft tissue stability.^3^, ^4^ Methods of internal fixation for ulnar coronoid fractures include Kirschner wires, tension screws (cancellous bone screws), mini plates, anchor and suture “lasso” techniques. For larger fractures, surgeons can choose lag screw fixation methods directed anterior to posterior or *vice versa*.^5^, ^6^, ^7^, ^8^ Since the proximal ulna is covered with thin cortex and loose spongiosa, especially in patients with osteoporosis, problems such as screw loosening, displacement or rotation of the fracture block, and screws stripping may occur when the single or plural screws are improperly fixed.^9^, ^10^, ^11^, ^12^ To avoid problems with a single screw, double lag screws sometimes can be used to enhance fixation stability. However, experimental studies have shown that there is no significant difference in lateral bending stability between single‐screw fixation and double‐screw fixation,^13^, ^14^ and the single‐screw fixation has the advantages of reducing time and costs of operation.

At present, it is still uncertain whether single screw has the same stability as double screws in the treatment of ulnar coronal process basal fracture (Regan–Morry type III ulnar coronoid fracture). The aims of this study were: (i) to measure the cancellous bone mineral density (BMD) and fixed length of the proximal end of the ulna and coronal process by three‐dimensional reconstruction of CT images of volunteers with normal elbow joints; (ii) to compare the pull‐out force and anti‐rotation torque of anterior single screw‐cancellous bone fixation (aSSBF) and anterior double screw‐cancellous bone fixation (aDSBF) in ulnar coronal process basal fracture by the sensitivity analysis of the finite element (FE) models; (iii) to further study the influencing factors of pull‐out stability and rotational stability of smart screw internal fixations in ulnar coronal process basal fracture.

## Materials and Methods

This research was approved by the Ethics Committee of Our Medical University (2020‐028).

### 3D Measurement of BMD and Fixed Length of the Proximal Ulna and Coronal Process

We collected CT images of 63 normal adult elbow joints from January 2016 to August 2019 at the Medical Examination Center of the First Affiliated Hospital of Our Medical University. The CT scan slices ranged from 0.5 to 1.0 mm and were saved in DICOM format. There were 41 cases in the young group aged 15–44 years, including 22 men and 19 women; 22 cases in the middle‐aged and younger elderly group aged 46–68 years, including seven men and 15 women. Inclusion criteria: well‐developed healthy adult volunteers, whose height, weight, and body mass index (BMI) meet the standards and have no upper limb deformity. Exclusion criteria: history of trauma (fracture at any part of the upper limb, malunion of fracture, joint dislocation or dislocation, etc.); congenital malformations such as ulna and radius fusion, achondroplasia, rickets, and gigantism; bone hyperplasia, porosity, degeneration, osteoarthritis and other bone diseases; joint diseases caused by systemic diseases such as diabetes, rheumatism or rheumatoid arthritis, hypertension, pregnancy, other endocrine diseases, etc.

**FIGURE 1:**
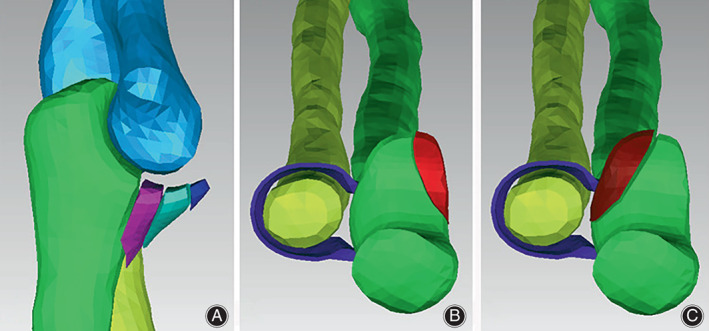
(A–C) 3D digital anatomy models of Regan–Morry classification of ulnar coronoid process fractures. (A) Regan–Morry type‐I, II, III; (B) Regan–Morry type‐IV‐M. (C) Regan–Morry type‐IV‐L

**FIGURE 2:**
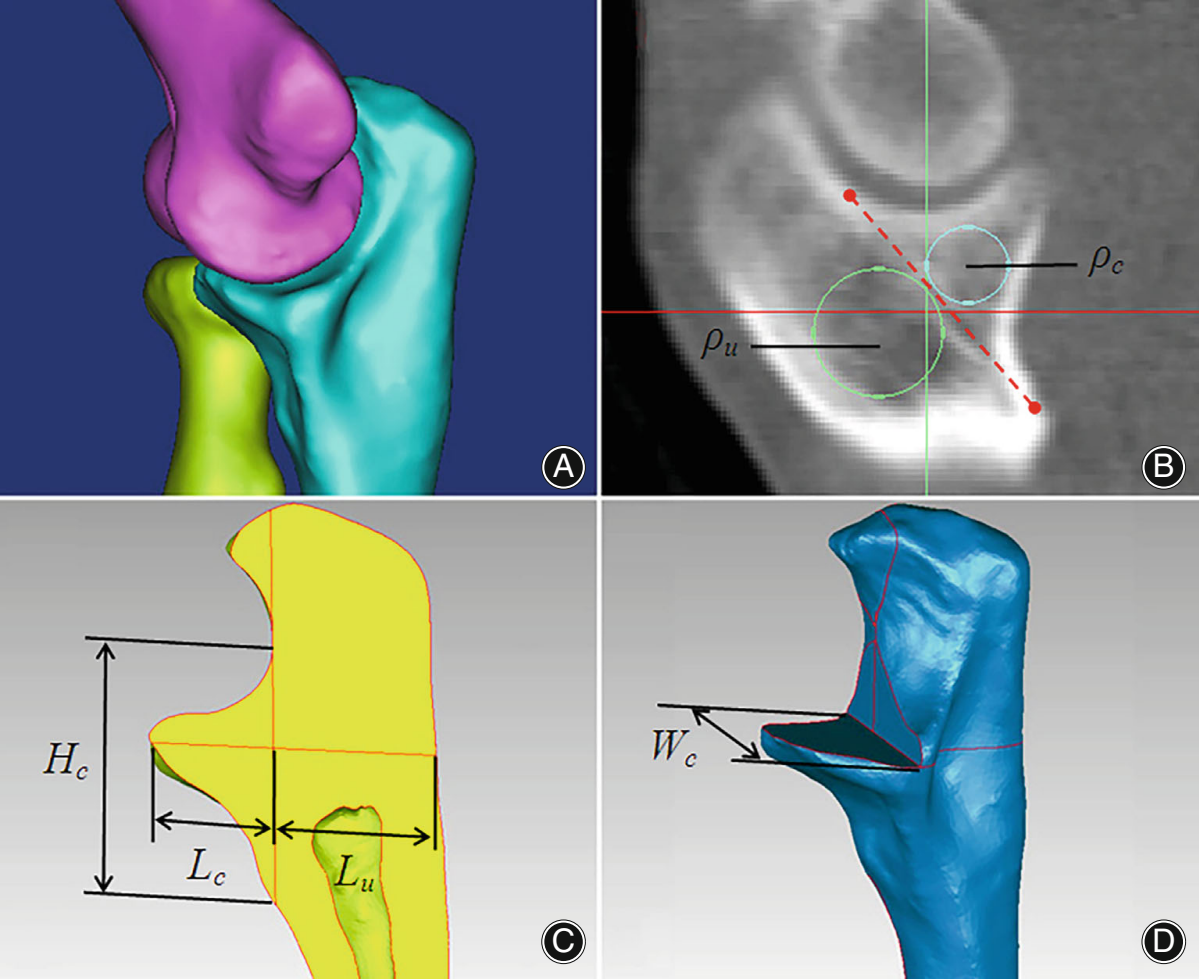
(A–D) Schematic diagram of the 3D measurement of the bone mineral density (BMD) and fixed length of the proximal ulna and the coronal process of the elbow. (A) 3D reconstruction of the elbow joint. (B) The BMD of the proximal ulna and the coronal process (*ρ*_u_, *ρ*_c_). (C) and (D) Proximal ulna sagittal diameter length (*L*_u_) and coronal process sagittal diameter length (*L*_c_), the longitudinal height of the base of the coronal process (*H*_c_) and transverse width (*W*_c_)

The 63 normal elbow‐joint CT images were imported into Mimics software (Materialise Company, Belgium) for 3D reconstruction. Then the 3D models were input into Geomagic software (Geomagic Company, USA) for sectioning and measurements. According to the Regan–Morry classification of ulnar coronoid process fracture, 3D digital anatomy models were built, as shown in Figure 1A–C. Aiming at the fracture of the base of the coronal process (Type III in Regan–Morry classification), the appropriate sagittal, coronal, and transverse planes of the 3D model of the ulna were captured, as shown in Figure 2A–D. The BMDs (*ρ*_u_, *ρ*_c_) of the proximal ulna and the coronal process located at the sagittal plane, proximal ulna sagittal diameter length (*L*_u_) and coronal process sagittal diameter length (*L*_c_), longitudinal height (*H*_c_) and lateral width (*W*_c_) at the base of the coronal process were measured as shown in Figure 2B–D.

### FE Modeling of Elbow Joint Posterior Dislocation with Coronoid Fractures

#### FE Model of Intact Elbow Joint and Forearm (IEJF)

**FIGURE 3:**
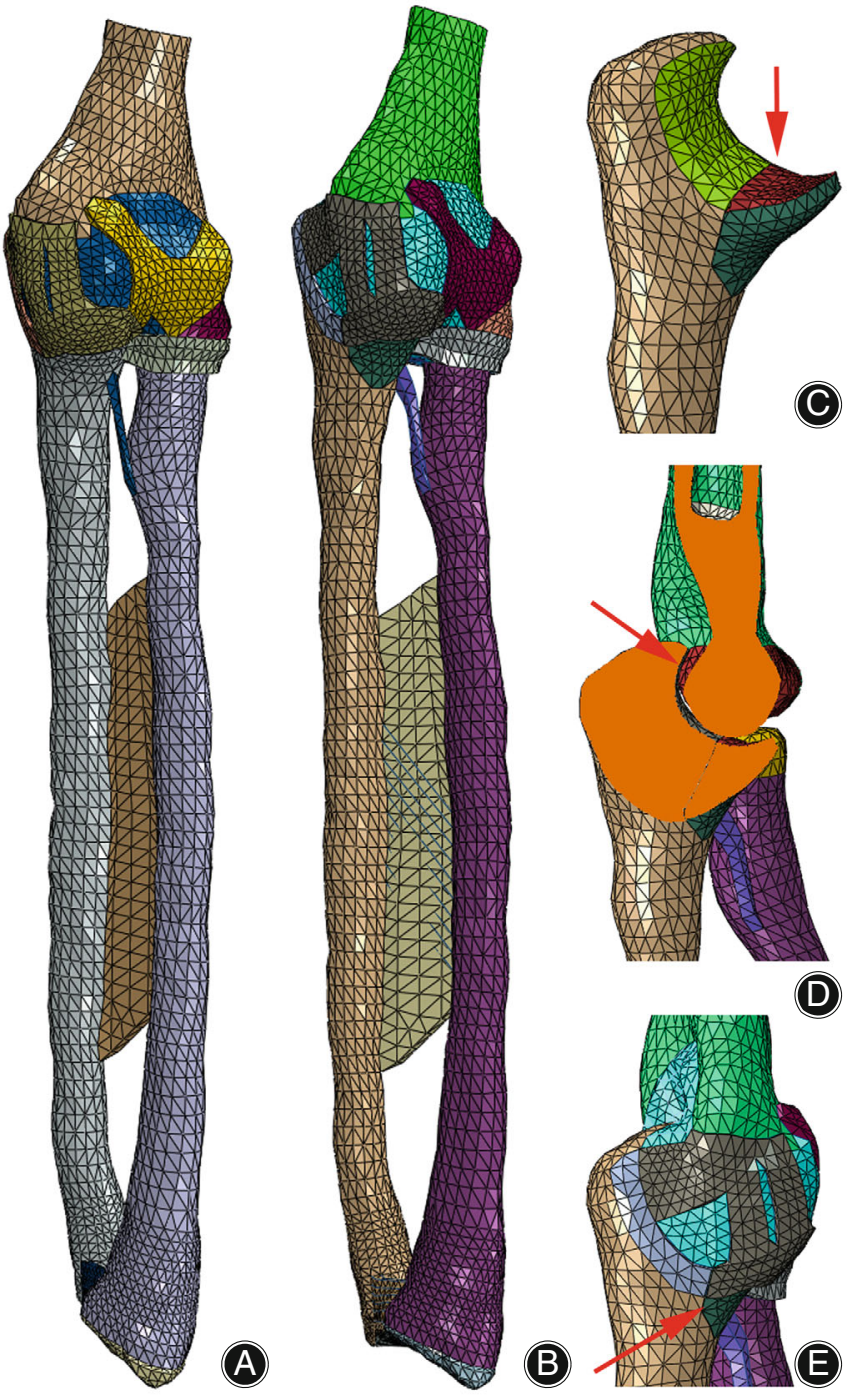
The finite element (FE) models of intact and fractured elbow joint and forearm. (A) The FE models of intact elbow joint and forearm; (B) The FE models of coronal process fracture and posterior dislocation of elbow joint; (C) Coronal process fracture (arrow); (D) Posterior dislocation of elbow joint (arrow); (E) Partial rupture of ulnar collateral ligament (arrow)

The FE modeling research adopts the CT and magnetic resonance (MR) image datasets of the second‐generation Chinese Digitized Human (F2‐CDH) “female No. 24” (the digitized model of a Chinese volunteer with standard body figure, female, 24 years old, height 167 cm, mass 51 kg, BMI 18.3, collected in 2007).^15^ The human body was simulated to maintain the attention position during scanning. The FE model of the elbow joint and forearm was constructed using Mimics software, Geomagic software, and Abaqus 6.14‐4 FE analysis software platform (Dassault Company, France), and the longitudinal space between the humeroradial and humeroulnar joints was within the normal value (Figure 3A). The distal humerus, ulna, and radius (including the cortical bone, cancellous bone, articular disc, and articular cartilage) were defined as solid elastic elements. The joint capsule matrices (such as those of humeroulnar joint, humeroradial joint, proximal radioulnar joint, distal radioulnar joint), the ligament matrices (such as those of anterior oblique ligament (AOL, two bundles), ulnar collateral ligament (UCL, three bundles), radial collateral ligament (RCL, three bundles), annular ligament (AL, one bundle), ulnar radial ligament (URL, one bundle), ulnar transverse collateral ligament (UTCL, one bundle), radioulnar oblique cord ligament (RUOL, one bundle), anterior distal radioulnar ligament (ADRUL, one bundle), posterior distal radioulnar ligament (PDRUL, one bundle)), forearm interosseous membrane (IOM) matrix, and other matrices were defined as solid hyperelastic material elements. Fibers of various joint capsules, interosseous membranes, and ligaments were defined as link cable elements. The humeroulnar articular contact, humeroradial articular contact, upper radioulnar articular contact, lower radioulnar articular contact, joint capsule contact with humeral cartilage, joint capsule contact with radial cartilage, joint capsule contact with humeral cartilage, joint capsule contact with humeral olecranon fossa dorsum, and articular disc contact with ulnar cartilage were defined as slidable plane to plane contact elements with a friction coefficient of 0.0024 to 0.24. The FE modeling parameters of the IEJF model are shown in Figure 3A, and the material properties of various tissues are provided in Table 1.^15^, ^16^, ^17^

**TABLE 1:** The material properties of various tissues of elbow‐forearm finite element (FE) model

Material	Element type	Elastic modulus *E* (Mpa)	Poisson ratio ν	Cross section area *A* (mm^2^)
Cortical bone	C3D4	13,400	0.3	‐
Cancellous bone	C3D4	325	0.29	‐
Cartilage/articular disc	C3D4	10	0.4	‐
Capsule matrix	C3D4	1.1	0.49	‐
Ligament matrix	C3D4	2.1	0.48	‐
IOM matrix	C3D4	2.1	0.3	‐
AOL fiber	T3D2	260	0.3	18.40 × 2
UCL fiber	T3D2	260	0.3	18.40 × 3
RCL fiber	T3D2	260	0.3	18.40 × 3
AL fiber	T3D2	260	0.3	18.40
URL fiber	T3D2	260	0.3	18.40
UTCL fiber	T3D2	260	0.3	18.40
RUOL fiber	T3D2	260	0.3	18.40
ADRUL fiber	T3D2	260	0.3	18.40
PDRUL fiber	T3D2	260	0.3	18.40
IOM fiber	T3D2	350	0.3	9.50 × 2
Capsule fiber	T3D2	105	0.3	445.47

*Note*: Matrix (Mooney–Rivlin hyperelasticity): *W* = C10(I1‐3) + C01(I2‐3), (C10+ C01) = *E*/4(1 + *ν*), *E* is elastic modulus, *ν* is Poisson ratio, I1 and I2 are the first and second invariants of the Cauchy‐Green tensor.^16^, ^17^

Abbreviation: ADRUL, anterior distal radioulnar ligament; AL, annular ligament; AOL, anterior oblique ligament; IOM, interosseous membrane; PDRUL, posterior distal radioulnar ligament; RCL, radial collateral ligament; RUOL, radioulnar oblique cord ligament; UCL, ulnar collateral ligament; URL, ulnar radial ligament; UTCL, ulnar transverse collateral ligament.

#### FE Model of Fractured Ulnar Coronoid Process and Forearm (FUCF)

**FIGURE 4:**
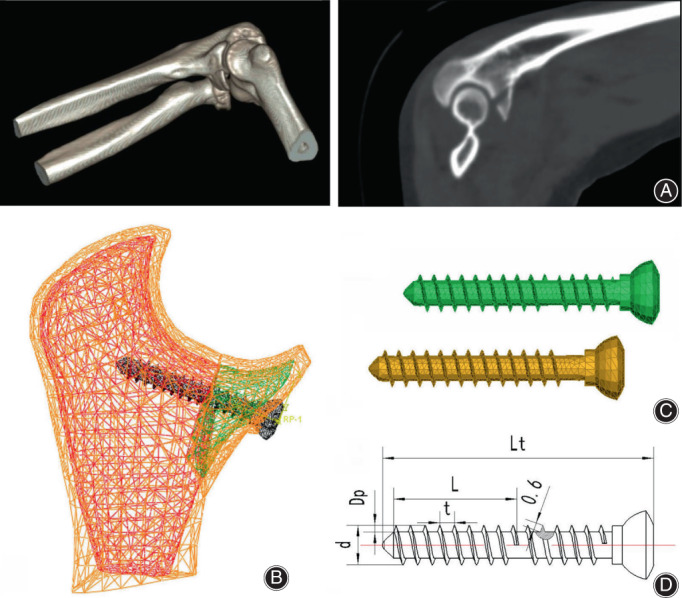
(A–D) Finite element (FE) modeling of ulnar coronoid fracture treated with the screw. (A) 3D‐CT of an ulna coronoid fracture case (female, 51 years old, Regan‐Morry III type). (B) Finite element (FE) model of aSSBF. (C), (D) Screw 3D models of aSSBF and aDSBF, screw cross section view with 3.5 mm diameter: *d* = 3.5 mm, Dp = 0.57 mm, *t* = 1.3 mm, *b* = 0.6 mm, *L* is screw fixed length, Lt is screw whole length, Dp is thread OD

In the normal ulna model, according to a fracture case of Regan–Morry type III (female, 51 years old, collected in March 2015, as shown in Figure 4A) and relevant clinical knowledge of ulnar coronoid process fractures,^5^ an FE model of ulnar coronoid process fracture (Regan–Morrey Type III) was constructed, which included three injury factors—coronoid process fracture, posterior dislocation of the elbow joint, and partial injury of the ulnar collateral ligament (UCL). In coronoid process fracture, the fractured surface was located at the bottom of the coronoid process base, the distance between the fractured surfaces was about 0.2 mm, and the friction coefficient was 0.24; two contact pairs were included, cortical bone and cancellous bone, which are defined as surface‐to‐surface contact elements (Table 1). In ulnar coronoid process fracture model, the fractured surface of the ulna together with the coronoid process offset backwards by about 3 mm, resulting in posterior dislocation of the elbow joint. In partial injury of the UCL, the ulnar collateral ligament was ruptured at the fractured surface of the coronoid process, resulting in the fracture of medial and lateral ligaments, while the other fibers were well‐attached. In this model, the radial head injury was not considered, so both the radial head and its adjacent ligaments were intact. The FE modeling parameters of the FUCF model are shown in Figure 3B–E, and the material properties of various tissues^15^, ^16^, ^17^ are shown in Table 1.

### Sensitivity Analysis of Pull‐out Force of FE Models of aSSBF and aDSBF

In the FUCF model (Figure 3B–E), a cancellous bone model of ulnar coronoid process was extracted. An FE mesh model of ulnar coronoid process fracture treated with aSSBF was established, among which the fractured surface was at the bottom of the coronoid base and the pore thread diameter of the cancellous bone was consistent with the screw model, as shown in Figure 4B. The screw was an international general‐purpose screw with a diameter of 3.5 mm, the screw parameters were measured by a 3D projection measuring instrument (TZTEK Company, China), and the 3D screw modeling was created by CATIA (Dassault Company，France), its geometric parameters and three‐dimensional screw models of aSSBF and aDSBF are shown in Figure 4C,D.

**FIGURE 5:**
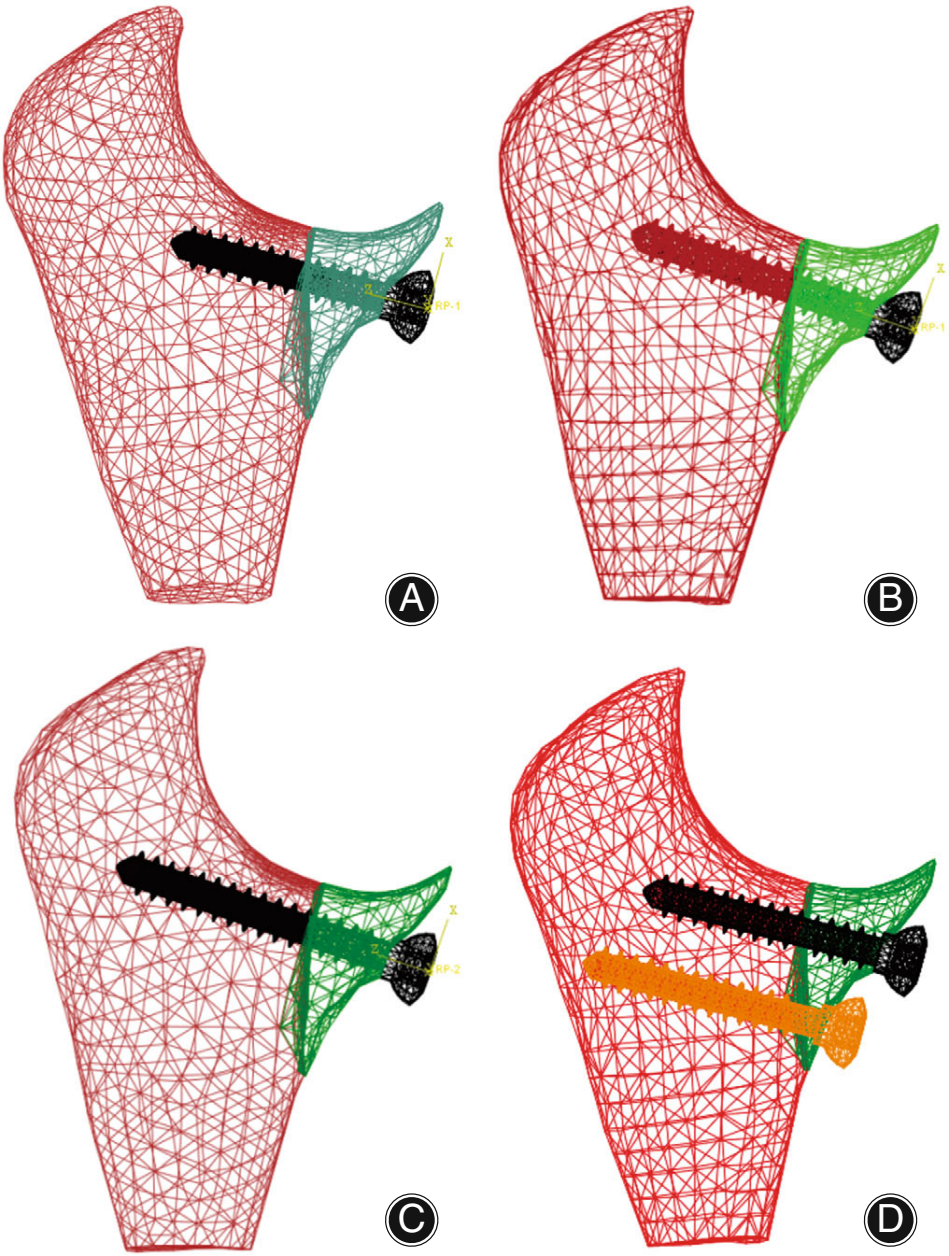
(A–D) Screw‐cancellous bone pull‐out force of finite element (FE) models of aSSBF and aDSBF. (A) aSSBF model with fixed length *L* = 9.4 mm. (B) aSSBF model with fixed length *L* = 12 mm. (C) aSSBF model with fixed length *L* = 14.6 mm. (D) aDSBF model with fixed length *L*_1_ = 12 mm and *L*_2_ = 16.5 mm

In ABAQUS software (Dassault Company, France), the anterior screw, ulnar cancellous bone and its coronoid fracture block were assembled, and the FE modeling was completed. The calculation model simplifies cortical bone as constraints, the minimum and maximum mesh sizes of cancellous bone mesh were set to 0.15 and 1.1 mm, respectively, its element type was defined as quadratic tetrahedron elements (C3D10M). The minimum screw grid was set to 0.3 mm, its element type was defined as primary tetrahedral element (C3D4). It was assumed that the cancellous bone material is homogeneous and isotropic. Three kinds of bone mineral density which represent different degrees of osteoporosis were used^18^ and their elastic modulus and yield strength were consistent with Bevill's research^19^ and Mercer's experiment.^20^ The material of the screw was set to titanium alloy (Ti6A14V), and the corresponding material parameters were shown in Table 2. The surface‐to‐surface contact relationship between the outer surface of the screw and the cancellous bone pore thread was established, and the friction coefficient was set to 0.2,^21^ but a shared node bond was established between the outer surface of the screw and the inner surface of coronoid fracture block. In view of the fact that the fixed length of the anterior screw would affect the pull‐out force, three kinds of single‐screw fixed lengths (9.4, 12, 14.6 mm) of aSSBF models were designed, as shown in Figure 5A–C: (A) 150,111 solid elements and 15,886 contact elements; (B) 201,129 solid elements and 20,626 contact elements; (C) 221,325 solid elements and 18,969 contact elements. A type of double‐screw fixed length (12, 16.5 mm, respectively) of aDSBF model was designed, as shown in Figure 5D: (D) 210,059 solid elements and 24,146 contact elements.

**TABLE 2:** Material parameters of the screw and cancellous bone

Materials	Density g/cm^3^	Tensile elastic modulus (MPa)	Tensile yield strength (MPa)	Poisson ratio	Shear modulus of elasticity (MPa)	Shear yield strength (MPa)
Screw	4430	110,000	860	0.3	42,307	‐
Bone	0.16 (low)	57	2.2	0.2	23	1.41
	0.24 (medium)	143	3.9	0.2	44	2.78
	0.32 (high)	267	5.9	0.2	67	3.89

In the numerical pull‐out test, the screw nut was rigidly coupled, and an axial load was applied to the center of the screw nut with a loading speed of 0.03 mm/s, four indexes are obtained when pulling out the anterior screw: (1) failure displacement (node displacement when the load does not increase with the displacement), (2) pull‐out force, (3) maximum von Mises stress of screw, and (4) maximum von Mises stress of cancellous bone.

### Sensitivity Analysis of Anti‐Rotation Force of FE Models of aSSBF and aDSBF

**FIGURE 6:**
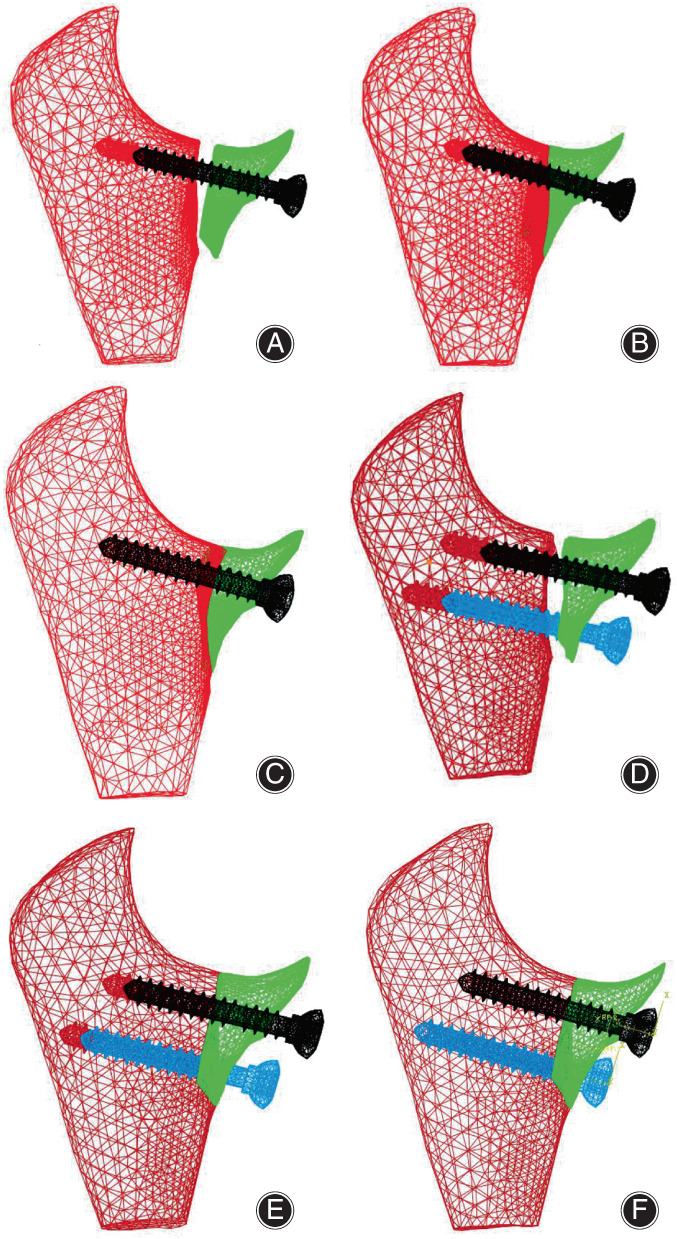
(A–F) Screw‐cancellous bone rotation torque of finite element (FE) models of aSSBF and aDSBF. (A), (D) the coronoid fracture block is bilaterally not in contact with the ulnar cancellous bone and the screw nut (BNC). (B), (E) the coronoid fracture block is attached to the ulnar cancellous bone, but unilaterally not in contact with the screw nut (UNC). (C), (F) the coronoid fracture block is bilaterally in contact with the ulnar cancellous bone and the screw nut simultaneously (BIC)

The anterior screw‐cancellous bone rotation model adopted the element types of the anterior screw‐cancellous bone pull‐out force model, but the meshes of the geometric contact area of the coronoid process were encrypted, with their minimum sizes reaching 0.15 mm. The material properties of cancellous bone were treated with a high‐density category (0.32 g/cm^3^),^18^ which were close to that of cancellous bone around the coronoid process. Three pairs of contact relationships between screws and ulnar cancellous bone, ulnar cancellous bone and coronoid fracture block, screw nut and coronoid fracture block were established, and their friction coefficients were set to 0.2.^21^ Simulating anatomical reduction state of fractured surface and screw fixation position, six FE models of screws‐cancellous bone rotation analysis for aSSBF and aDSBF were established, as shown in Figure 6A–F: (A) and (D) show that the coronoid fracture block is bilaterally not in contact with the ulnar cancellous bone and the screw nut (bilaterally non‐contact, BNC, (A) contains 61,646 solid elements and 2190 contact elements, (D) contains 64,784 solid elements and 4716 contact elements); (B) and (E) indicate that the coronoid fracture block is attached to the ulnar cancellous bone, but unilaterally not in contact with the screw nut (unilaterally non‐contact, UNC, (B) contains 61,646 solid elements and 5572 contact elements, (E) contains 64,784 solid elements and 6707 contact elements); (C) and (F) show that the coronoid fracture block is bilaterally in contact with the ulnar cancellous bone and the screw nut simultaneously (bilaterally in contact, BIC, (C) contains 61,646 solid elements and 6412 contact elements, (F) contains 71,307 solid elements and 7801 contact elements). In the above models, BNC is of poor fitting degree, UNC is of average moderate degree, and BIC is of good fitting degree.

For the rotation models with both sides in contact (BIC), three different pre‐tightening loads (PTL) states were set: PTL = 0 N, PTL = 20 N, and PTL = 40 N, which could tighten cancellous bone screw appropriately,^12^ and act as the prestressed concrete to preserve the prestress in cancellous bone in the fracture zone. The calculation model simplified peripheral cortical bone as constraints, the axial rotational angular displacement of the screw was applied to the fracture block. According to outcomes of preliminary experiments, failure rotation angle of the coronoid fracture block is about 5° to 15° (among this range the ulnar cancellous bone surrounding the screw yield). The limiting angle of the single screw fixation rotation test was set up for about 15°, and the limiting angle of the double screws fixations rotation test was about 5°.

In this numerical rotation test, four indexes were obtained when the anterior screw was rotated at failure state: (1) ultimate torque (torque load at the limit of the rotation angle); (2) screw cross section load (screw axial working load in cross section at the limit rotation angle); (3) the maximum von Mises stress of screw; (4) the maximum von Mises stress of cancellous bone.

### Validations of FE Models

The biomechanical experiments by Renani et al.^22^ reported that with the action of axial forces of 80, 110, and 140 N when an intact elbow flexed at about 20° to the forearm, the maximum contact stress of the humeroulnar joint (coronoid process) was 0.64 ± 0.1 to 1.41 ± 0.25 MPa, which changed linearly. In this study, when axial forces (50, 100, and 150 N) were applied under the same experimental conditions, the IEJF FE model (Figure 3A) predicted that the maximum contact stress of the humeroulnar joint (coronoid process) was 0.41–1.07 MPa (0.61–1.01 MPa under the condition of equal proportional load with the experiment), which changed linearly; meanwhile, the aSSBF and aDSBF models predicted that the maximum contact stress of the humeroulnar joint (coronoid process) was 0.44–1.08 MPa (0.63–1.02 MPa under the condition of equal proportional load with the experiment), which changed linearly. The experimental values were close to the calculated values.

According to the biomechanical experiment by Hackl et al.^23^ under the axial compression of 50, 100, and 150 N, the stress ratios of the humeroradial joint (radial head) were 62.9% ± 25.8%, 61.9% ± 16.8%, and 66.5% ± 16.1%, respectively. In this study, under the same experiment conditions of axial compression (50, 100, and 150 N), the IEJF FE model (Figure 3A) predicted that the stress ratios of the humeroradial joint (radial head) were 64.4%, 61.2%, and 60.9%, respectively. The experimental values were consistent with the calculated values.

In addition, under the same axial compression experiment conditions (50, 100, and 150 N), the fractured and dislocated elbow joint FE model (Figure 3B–E) predicted that the stress ratios of humeroradial joint (radial head) were 0.01%, 7.0%, and 13.9%, respectively. The radial head lost most of the stress, which was basically consistent with the clinical experience.

**FIGURE 7:**
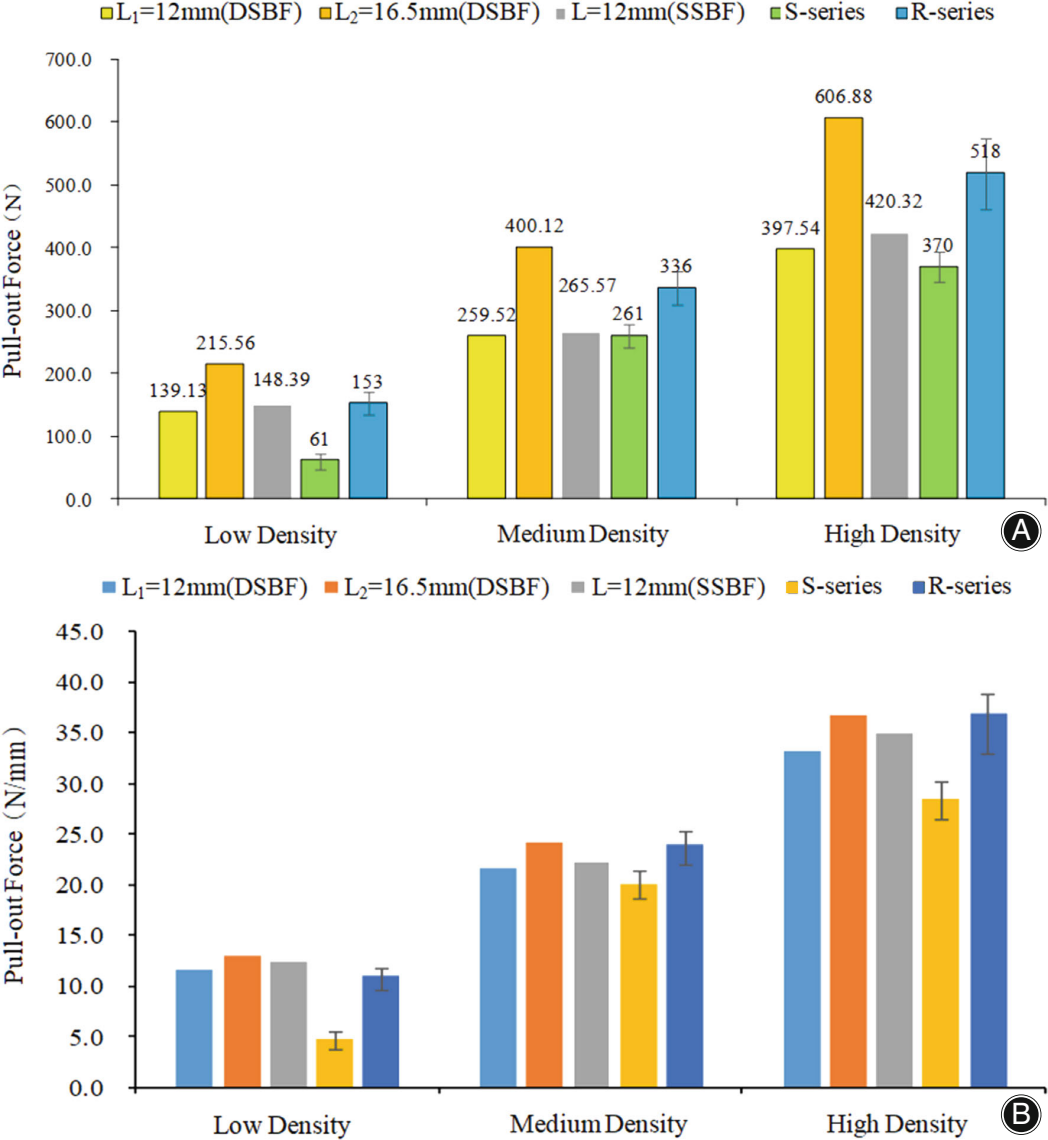
(A and B) The validations of screw‐cancellous bone finite element (FE) models. (A) the comparisons of the model‐predicted and experiment‐measured pull‐out loads. (B) the comparisons of the model‐predicted and experiment‐measured pull‐out loads under the same fixed length inside bone

In the study of low, medium, and high bony density,^18^, ^24^ the comparison between the numerical results of pull‐out force of SSBF or DSBF model and the experimental results of pull‐out load of S‐series or R‐series screw fixation reported by Chapman et al.^25^ were shown in Figure 7A. The FE model‐predicted pull‐out forces compared to those of S‐series and R‐series screws experimental results under the same fixed length of inside bone, the two kinds of load values (numerical and experimental values) are close to each other, as shown in Figure 7B. The screw parameters are shown in Table 3.^25^ Therefore, the pull‐out forces calculated in our FE models corresponded to those determined previously in biomechanical experiments.

**TABLE 3:** Parameter table of the screw model and screw physical object

Screw index	Screw model 1	Screw model 2	S205. 40 physical screw	R121840 physical screw
Diameter (mm)	3.5	3.5	3.5	4
Length inside bone (mm)	12	16.5	13	14
Pitch (mm)	1.3	1.3	1.25	1.75
Thread OD (mm)	0.57	0.57	0.5	0.63

*Note*: Thread OD, thread outer diameter

### Statistical Analysis

All data management and analysis were conducted using IBM SPSS Statistics for Windows version 20.0 (IBM SPSS Inc., Chicago, Illinois, USA). The statistical outcomes of bone mineral densities, geometric structural parameters of proximal ulna and coronoid process were expressed as mean ± standard deviation (SD). The multivariate analysis of variance in the general linear model program was used to compare the differences of bone mineral densities, geometric structural parameters of proximal ulna and coronoid process in the sex and age categories. *p* < 0.05 was considered as the differences that achieved statistically significant level.

## Results

### The BMD and Fixed Length of Proximal Ulna and Coronal Process

The BMD and fixed length of the proximal ulna and coronal process of 63 volunteers are shown in Table 4. The difference of *ρ*_c_ between sex is not significant (*p* = 0.610), but for age, the difference is significant (*p* = 0.013); the difference of *ρ*_u_ between sex (*p* = 0.230) and age (*p* = 0.221) are not significant; the differences of *L*_c_, *L*_u_, *W*_c_ and *H*_c_ between sex are significant (*p* < 0.001, *p* < 0.001, *p* < 0.001, *p* = 0.013), but the differences between age are not significant (*p* > 0.05). According to the measured value of BMD, it can be classified as three types of bone density in the calculation: low density 0.16 g/cm^3^, medium density 0.24 g/cm^3^, and high density 0.32 g/cm^3^. According to Regan–Morry type III, the measured values of the proximal ulnar and coronal process sagittal diameter length, and the height and width of the coronal process base, two types of screw fixation length calculation types can be defined: aSSBF (12 mm), aDSBF (12, 16.5 mm).

**TABLE 4:** The bone mineral density (BMD) and fixed length of proximal ulna and coronal process

Observation index	Age		Factor source	Variance	SS	*F* value	*p* value
	Young group	Elderly group					
*ρ*_*c*_: female (g/cm^3^)	0.300 ± 0.026	0.277 ± 0.041	Sex	1	0.000	0.262	0.610
*ρ*_*c*_: male (g/cm^3^)	0.307 ± 0.038	0.281 ± 0.035	Age	1	0.008	6.548	0.013
			Sex × age	1	0.000	0.036	0.851
*ρ*_*u*_: female (g/cm^3^)	0.171 ± 0.025	0.167 ± 0.038	Sex	1	0.000	1.473	0.230
*ρ*_*u*_: male (g/cm^3^)	0.187 ± 0.028	0.171 ± 0.033	Age	1	0.001	1.532	0.221
			Sex × age	1	0.000	0.448	0.506
*L*_*c*_: female (mm)	15.128 ± 1.248	15.697 ± 1.851	Sex	1	59.399	27.131	0.000
*L*_*c*_: male (mm)	17.189 ± 1.425	17.336 ± 1.323	Age	1	5.423	2.477	0.121
			Sex × age	1	0.075	0.034	0.853
*L*_*u*_: female (mm)	16.679 ± 1.237	17.409 ± 1.127	Sex	1	82.847	20.640	0.000
*L*_*u*_: male	19.204 ± 2.960	19.932 ± 1.117	Age	1	6.908	1.721	0.195
			Sex × age	1	0.000	0.000	0.999
*W*_*c*_: female (mm)	20.145 ± 1.857	21.416 ± 2.575	Sex	1	127.569	14.459	0.000
*W*_*c*_: male (mm)	23.173 ± 3.938	24.653 ± 2.580	Age	1	24.607	2.789	0.100
			Sex × age	1	0.141	0.016	0.900
*H*_*c*_: female (mm)	30.554 ± 2.508	30.479 ± 3.322	Sex	1	53.742	6.537	0.013
*H*_*c*_: male (mm)	31.902 ± 2.761	33.198 ± 3.091	Age	1	4.846	0.589	0.446
			Sex × age	1	6.115	0.744	0.392

*Note*: *ρ*_c_ cancellous bone density in the coronal process area, *ρ*_u_ cancellous bone density of the proximal ulna posterior to the coronal process, *L*_c_ sagittal length of the coronal process, *L*_u_ sagittal length of the proximal ulna posterior to the coronal process, *W*_c_ transverse width of the coronal process, *H*_c_ longitudinal height of the coronal process

### Pull‐out Forces and Stresses of aSSBF and aDSBF

The pull‐out force and the failure displacement of aSSBF model and aDSBF model on the BMD, and the fixed length of the screw are shown in Table 5. (1) The pull‐out failure displacement of a single screw decreases with the increase of bone density but increases with the depth increase of the screw. (2) The pull‐out load of a single screw is positively correlated with the density of the cancellous bone and is highly linearly related to the screw fixed depth. (3) The pull‐out load of the double screws is not a linear addition of two single screws but is close to the sum of the pull‐out loads of two single screws with the same fixed depths corresponding to the double screws. It is equivalent to the effect of increasing the fixed length or the outer diameter of a single screw.

**TABLE 5:** The pull‐out forces and pull‐out stresses of aSSBF and aDSBF

Model	Bone density (g/cm^3^)	Screw fixed length (mm)	Failure displacement (mm)	Pull‐out force (N)	Maximum screw stress (Mpa)	Maximum stress of cancellous bone (Mpa)
(aSSBF)	0.16 (low)	*L* = 12	0.41	148.39	47.32	2.2
	0.24 (medium)	*L* = 12	0.32	265.57	83.11	3.9
	0.32 (high)	*L* = 12	0.25	420.32	129.90	5.9
	0.32	*L* = 9.4	0.24	315.59	95.56	5.9
	0.32	*L* = 12	0.25	420.32	129.90	5.9
	0.32	*L* = 14.6	0.26	530.59	155.80	5.9
(aDSBF)	0.16	*L*_1_ = 12	>0.40	139.13	54.05	2.2
	0.16	*L*_2_ = 16.5	0.40	215.56	94.59	2.2
	0.24	*L*_1_ = 12	>0.29	259.52	98.35	3.9
	0.24	*L*_2_ = 16.5	0.29	400.12	153.20	3.9
	0.32	*L*_1_ = 12	>0.24	397.54	136.00	5.9
	0.32	*L*_2_ = 16.5	0.24	606.88	215.80	5.9

Abbreviations: aDSBF, double screw‐cancellous bone fixation; aSSBF, anterior single screw‐cancellous bone fixation.

As seen from Table 5, (1) the maximum stress of the screw located at the root of the screw nut is positively related to the bone density and screw fixed depth, and the single screw stress is slightly less than that of the double screws. (2) The maximum stress value of cancellous bone is positively correlated with bone density, but it has nothing to do with the screw fixed depth. The maximum stress is located at the root of the bone screw thread. When the single screw or the double screws are pulled out, the maximum stresses of cancellous bones in two pull‐out tests are comparable and by that time all cancellous bones have yielded.

### Anti‐Rotating Torques and Stresses of aSSBF and aDSBF

The ultimate torque and load of aSSBF model and aDSBF model under different reset and pre‐tightening conditions are shown in Table 6. (1) Under BNC condition, aSSBF model has a relatively small and constant torque generated by the frictions between screw thread and cancellous bone. Under UNC condition, the ultimate torque increases by 18.6‐folds. Under BIC condition, the anti‐rotation torque continues to increase, while the pre‐tightening load gradually intensifies the anti‐rotation torque. (2) The fixation system of double screws still has a high ultimate torque when bilateral sides are not fitted (BNC). The ultimate torque slightly increases when the fractured surface is unilaterally attached (UNC). The anti‐rotation torque continuously increases when the fractured surface and screw nut are bilaterally attached (BIC). Similar to single screw fixation, the pre‐tightening load can gradually and slightly intensify the anti‐rotation torque of aDSBF model.

**TABLE 6:** Rotational torques and stresses of aSSBF and aDSBF under different pre‐tightening loads (PTL)

Model	Contact status and PTL (N)	Ultimate torque (N mm)	Screw cross section load (N)	Maximum stress of screw (Mpa)	Maximum stress of cancellous bone (Mpa)
(aSSBF) Rotating 15°	(BNC)	6.64	0	3.93	1.76
	(UNC)	130.05	0	99.97	5.9
	(BIC), PTL = 0	168.64	40.28	126.70	5.9
	(BIC), PTL = 20	178.90	50.77	135.00	5.9
	(BIC), PTL = 40	183.83	56.82	143.90	5.9
(aDSBF) Rotating 5°	(BNC)	304.19	0	123.70	5.9
	(UNC)	311.81	0	63.61	5.9
	(BIC), PTL = 0	433.18	34.17	85.45	5.9
	(BIC), PTL = 20	442.61	44.30	86.01	5.9
	(BIC), PTL = 40	456.70	58.33	87.08	5.9

Abbreviations: aDSBF, double screw‐cancellous bone fixation; aSSBF, anterior single screw‐cancellous bone fixation; BNC, bilaterally non‐contact; PTL, pre‐tightening loads; UNC, unilaterally non‐contact.

Table 6 shows that: (1) Under BNC condition, the screw maximum stress in aSSBF model is minimal, while that in aDSBF model is obviously at a high level. Under UNC condition, screw maximum stress in aSSBF model increases quickly, but that in aDSBF model falls rapidly. Under BIC condition, screw maximum stresses of both models are in higher levels. With the increase of pre‐tightening loads, screw maximum stresses of both models increase slightly. (2) Under BNC condition, the stress of cancellous bone of aSSBF model is very small. In addition to this, the maximum stress of cancellous bone is comparable and yielded when aSSBF model and aDSBF model are rotating at their limit angles.

## Discussion

### Main Findings of this Study

The present study found and confirmed that fracture surface fitting degree and nut fitting degree are the other two important anatomical and biomechanical rotational stability factors of screw fixations for Regan–Morry type III ulnar coronoid fractures in adults. Then, three existing biomechanical pull‐out stability factors (bone density, screw fixation length, screw outside diameter and thread shape) were added to form the “five‐factor of anatomical and biomechanical stability of screw fixation”. When total five main stability factors meet reasonable conditions simultaneously, single or double screw fixation methods are stable for the treatments of ulnar coronoid basal fractures in adults.

### The Change Pattern of Pull‐out Forces of aSSBF and aDSBF for Pull‐out Stability

The pull‐out force of a cancellous bone screw is an important index for predicting screw loosening and fracture block displacement.^9^, ^11^, ^12^, ^21^ The study of Chatzistergos et al.^26^ has shown that the main parameters affecting the pull‐out force are the outer diameter of the screw and the fixed length inside bone, and the pull‐out force is linearly related to the fixed length of the bone. The experiments of Feng et al.^27^ indicated bone screws with a reverse buttress thread design will significantly increase the pull‐out strength. Chapman et al.^25^ studied the pull‐out force of S‐series and R‐series screws under artificial cancellous bone with different densities. It has been found that pull‐out force is positively correlated to bone density. In this study, bone density measurements of the proximal ulnar bone in 63 volunteers reveal that the bone density in the coronoid region of young adult men is close to the high‐density value of artificial cancellous bone (0.32 g/cm^3^), while the BMD of the ulnar bone posterior to the coronoid region of middle‐aged and elderly women is close to the low‐density value of artificial cancellous bone (0.16 g/cm^3^).^18^ Our study suggests that the pull‐out force of fully threaded screws (3.5 mm small diameter, 4.65 mm large diameter, 1.2 mm pitch) of the anterior single screw fixation is closely related to the fixed depth of the screw and bone mineral density, which is consistent with the findings of researchers such as Chatzistergos et al. and Chapman et al.^25^, ^26^ The pull‐out force of anterior double screw fixation is basically similar to that of anterior single screw fixation, and its pull‐out load (minimum force 139.13 and 215.56 N) is close to the sum of two single screw pull‐out loads under the same fixed depth (minimum force 148.39 × 2 N), which is equivalent to the effect of increasing the fixed length of a single screw, or the effect of increasing the outer diameter and changing the thread shape of a single screw.^26^, ^27^

### The Change Pattern of Anti‐Rotating Torques of aSSBF and aDSBF for Rotational Stability

The anti‐rotation torque of a cancellous bone screw is another important index for predicting screw rotation and rotational displacement of fracture block.^10^, ^12^ According to literature by Sasso et al.^14^ and Fang et al.^13^ two screws are commonly considered in clinical practice to enhance the lateral bending stability and rotational stability of the fracture block. The experiments of Xu et al.^12^ showed that “appropriate tightening cancellous bone screws” can avoid depression of the compression performance and loss of stability due to screw stripping. Our study has found that the ultimate rotation torque of the double screw fixation is indeed higher than that of the single screw fixation. But treating with a larger coronoid fracture block, if fracture reset is satisfactory and the screw nut is fitted close to the fracture block, single screw fixation can also satisfy the rotation stability of fracture reduction. This finding is in response to the rotation stability of the experimental study reported by Sasso et al.^14^ and Fang et al.^13^ Another interesting finding is that, regardless of anterior single screw fixation or anterior double screws fixations, the increase of pre‐tightening loads has little effect on the ultimate rotation torque. However, the pre‐tightening loads do create a prestress on the initial fracture zone, similar to prestressed concrete, which enhances the screw's pull‐out resistance. This is consistent with the experiment of Xu et al.^12^ and also meets the requirements of smart internal fixation.^28^

### Five‐Factor of Anatomical and Biomechanical Stability of Smart Screw Fixation

In this study, based on the existing three pull‐out stability factors (bone density, screw fixation length, screw outside diameter and thread shape, which were validated by our pull‐out force tests),^25^, ^26^, ^27^, ^28^ two new anatomical and biomechanical rotational stability factors (fracture surface fitting degree, nut fitting degree, which were confirmed by our anti‐rotation torque tests) were confirmed. Therefore, there are five main factors affecting the pull‐out force and anti‐rotation torque of screw fixations: bone density, length of screw fixation, outside diameter and thread shape of the screw, degree of fracture surface fit, and degree of nut fit, which is called the “five‐factor anatomical and biomechanical stability of screw fixation”. Although biomechanical stability of internal fixation of the two types of screws in fractures at the base of the coronoid process (type III of Regan–Morry fracture) is mainly studied in this paper, other Regan–Morry fractures, such as type II, type IV‐M, and type IV‐L (Figure 1A–C), all follow the “five‐factor anatomical and biomechanical stability of screw fixation”. Their pattern of biomechanical changes in pull‐out force and rotational moment resistance is essentially similar to that of Regan–Morry type III, with magnitudes showing similar proportionality. When the five main stability factors meet reasonable conditions simultaneously, the two types of screw fixation methods, namely anterior single‐screw fixation and anterior double‐screw fixation, are both stable for treatments of ulnar coronoid fractures.

### Strengths and Limitations

In this study, in addition to studying the pull‐out stability of the single screw and double screw fixation of the FE models, we also studied the rotational stability of the single screw and double screw fixation of the FE models and confirmed two important factors of anatomical and biomechanical stability of the rotational stability.

Although the simulation results in this paper can be well‐verified, the FE models of aSSBF and aDSBF in this paper still have some reasonable simplifications. The FE models of aSSBF and aDSBF have been simplified to some extent, and the minor influence of the ulnar ligaments on pull‐out force has not been taken into account. When defining bone materials as continuum materials,^29^ we only defined the elastic and plastic properties, but not the fracture parameters, which are closely related to bone density and can slightly increase the calculation accuracy in the fracture phase of the trabecular bone.

### Conclusions

Based on the existing three biomechanical pull‐out stability factors of screw fixations (bone density, screw fixation length, screw outside diameter and thread shape), two new anatomical and biomechanical rotational stability factors (fracture surface fitting degree, nut fitting degree) were confirmed and added to form the “five‐factor anatomical and biomechanical stability of screw fixation”. When the five main stability factors meet reasonable conditions simultaneously, aSSBF or aDSBF methods are stable for the treatment of ulnar coronoid basal fractures.

#### Authorship Declaration

All authors listed meet the authorship criteria according to the latest guidelines of the International Committee of Medical Journal Editors.

All authors are in agreement with the manuscript.

## Author Contributions

Hao Ye: Finite element analysis, data curation, model validation, funding acquisition. Yongchao Yang: case collected, model validation, clinical guidelines, resources. Tingyang Xing: Data curation, statistical analysis, model validation. Guirong Tan: Digital anatomy, 3D measurement, statistical analysis. Shuxun Jin: 3D measurement, statistical analysis. Zhichao Zhao: 3D measurement, statistical analysis. Weikang Zhang: 3D measurement, statistical analysis. Yanyan Li: 3D measurement, statistical analysis, funding acquisition. Lei Zhang: case collected, model validation, clinical guidelines, resources. Jianshun Wang: case collected, model validation, clinical guidelines, resources. Rongmei Zheng: 3D measurement, project administration. Yun Lu: Case collected, model validation, clinical guidelines. Lijun Wu: Conceptualization, methodology, investigation, finite element analysis, Writing—original draft, visualization, resources, funding acquisition, supervision.

## Funding Information

This work is supported by the National Natural Science Foundation of China (Grant no.: 81271663, 31471146), Wenzhou Medical University Scientific Development Foundation of Zhejiang, China (Grant no.: QTJ06012), Basic Scientific Research Project of Wenzhou City of China (Grant No.: Y20210398), Zhejiang Province Science and Technology Plan Research and Xinmiao Talent Program, China (Grant No.: 2021R413036).

## Conflict of Interest

The authors declare that they have no conflict of interest.

## Ethical Statement

This research was approved by the Ethics Committee of Wenzhou Medical University (2020‐028).
